# Gene expression profiling identifies candidate biomarkers for active and latent tuberculosis

**DOI:** 10.1186/s12859-015-0848-x

**Published:** 2016-01-11

**Authors:** Shih-Wei Lee, Lawrence Shih-Hsin Wu, Guan-Mau Huang, Kai-Yao Huang, Tzong-Yi Lee, Julia Tzu-Ya Weng

**Affiliations:** Taoyuan General Hospital, Ministry of Health and Welfare, Taoyuan, Taiwan; Department of Life Sciences, National Central University, Taoyuan, Taiwan; Institute of Medical Sciences, Tzu Chi University, Hualien, Taiwan; Department of Computer Science and Engineering, Yuan Ze University, Taoyuan, Taiwan; Innovation Center for Big Data and Digital Convergence, Yuan Ze University, Taoyuan, Taiwan

**Keywords:** Tuberculosis, Latent infection, Gene expression, Biomarker

## Abstract

**Background:**

Tuberculosis (TB) is a serious infectious disease in that 90 % of those latently infected with Mycobacterium tuberculosis present no symptoms, but possess a 10 % lifetime chance of developing active TB. To prevent the spread of the disease, early diagnosis is crucial. However, current methods of detection require improvement in sensitivity, efficiency or specificity. In the present study, we conducted a microarray experiment, comparing the gene expression profiles in the peripheral blood mononuclear cells among individuals with active TB, latent infection, and healthy conditions in a Taiwanese population.

**Results:**

Bioinformatics analysis revealed that most of the differentially expressed genes belonged to immune responses, inflammation pathways, and cell cycle control. Subsequent RT-PCR validation identified four differentially expressed genes, *NEMF*, *ASUN*, *DHX29*, and *PTPRC*, as potential biomarkers for the detection of active and latent TB infections. Receiver operating characteristic analysis showed that the expression level of *PTPRC* may discriminate active TB patients from healthy individuals, while *ASUN* could differentiate between the latent state of TB infection and healthy condidtion. In contrast, *DHX29* may be used to identify latently infected individuals among active TB patients or healthy individuals. To test the concept of using these biomarkers as diagnostic support, we constructed classification models using these candidate biomarkers and found the Naïve Bayes-based model built with *ASUN*, *DHX29*, and *PTPRC* to yield the best performance.

**Conclusions:**

Our study demonstrated that gene expression profiles in the blood can be used to identify not only active TB patients, but also to differentiate latently infected patients from their healthy counterparts. Validation of the constructed computational model in a larger sample size would confirm the reliability of the biomarkers and facilitate the development of a cost-effective and sensitive molecular diagnostic platform for TB.

**Electronic supplementary material:**

The online version of this article (doi:10.1186/s12859-015-0848-x) contains supplementary material, which is available to authorized users.

## Background

Tuberculosis (TB) is an infectious disease caused by various strains of mycobacteria, with *Mycobacterium tuberculosis* (*Mtb*) being the most common causative agent [1]. It is a serious global health threat with one-third of the world’s population estimated to be latently infected with *Mtb* [2]. Though about 90 % of those infected with *Mtb* are asymptomatic, possessing only a 10 % lifetime chance of developing active TB [3], even in developing countries with established healthcare systems, TB is still a deadly disease.

In 2006, the World Health Organization launched a “Global Plan to Stop Tuberculosis” that aims to save 14 million lives from TB by 2015. This objective is being hampered by the increase in HIV-associated tuberculosis and the emergence of multiple drug-resistant tuberculosis (MDR-TB) [4]. The only currently available vaccine is bacillus Calmette–Guérin (BCG) [5]. The vaccine is often administered to children, but the effectiveness of protection decreases after about 10 years.

With TB being one of the most common causes of death from infectious diseases, the current challenge is developing a sensitive and efficient method for the detection of latent TB infection (LTBI). The disease begins in the lungs via infection from the blood stream or aerosol droplets [6]. After TB bacteria enter the bloodstream, they can spread throughout the body and infect various tissues [7], such as the heart skeletal muscles, pancreas, or thyroid [8]. However, in LTBI, the bacteria remain dormant for several years before producing active TB. Even after treatment, the affected individual may still be susceptible to reactivation due to immunosuppression, or multiple-drug resistance in TB bacteria [4].

Substantial gene expression studies have revealed differences in the transcriptome between healthy controls and active TB or LTBI patients [9–11]. These findings not only uncovered important genetic signatures indicative of active TB and LTBI, but also identified transcriptionally regulated markers that are diverse in functions. In particular, these candidate genes are responsible for various key biological processes including inflammatory responses, immune defense, cell activation, homeostatic processes, regulation of cell proliferation and apoptosis. Moreover, these studies demonstrated the importance of cytokine and chemokine responses in the progression from latent infection to active disease [12–14]. However, the overall gene expression array results vary due to diverse genetic background of the study population and differences in the study design.

Early diagnosis of TB is crucial for preventing its spread, but the detection of LTBI is a major challenge as the carriers are often asymptomatic. Sputum smear acid-fast staining, though fast and inexpensive, is not the most sensitive and specific diagnostic test. While the tuberculin skin test represents a common diagnostic method, it has a tendency to produce false-positive results in individuals previously inoculated with BCG [15]. Culturing of TB bacteria usually takes time and diagnosis based on the test results is not always accurate. The interferon gamma release assays (IGRA) seem to have the potential of becoming the gold standard for TB test. The assays have been introduced into clinical practice to measure the amount of interferon-gamma (IFN-γ) released by blood cells infected wtih *Mtb* [16]. Unfortunately, this method is more expensive and requires blood samples with normal levels of viable leukocytes, which is not always possible in immunocompromised individuals. Consequently, an alternative quantitative polymerase chain reaction method was developed to detect the immune response to TB infection [17]. Yet, as most gene expression study results suggest, genetic background may influence the specificity and sensitivity of diagnosis.

Recently, Lu et al. (2011) conducted a gene expression microarray study to investigate the possibility of using mRNAs as biomarkers to differentiate active TB from LTBI [18]. Interestingly, in their study, the expression of IFN-γ, the biomarker used in IGRA, was not significantly different between the active TB and LTBI group [18]. Instead, the combination of three genes, *CXCL10* (chemokine C-X-C motif ligand 10), *ATP10A* (ATPase, class V, type 10A) and *TLR6* (toll-like receptor 6) appeared to be effective at distinguishing between active and latent TB infection. In contrast, *IL-8* (Interleukin 8), *FOXP3* (forkhead box P3), and *IL-12β* (interleukin 12 beta) were demonstrated to be the best discriminating biomarkers for TB and LTBI by Wu et al. [14]. Discrepancies between the two studies may be attributable to the differences in genetic background. At the same time, these findings suggest that not only are gene expression biomarkers more significant indicators of active TB, but they may also represent a more sensitive detection method for LTBI. Nevertheless, the same combination of genetic markers may not be applicable in another population.

For the present study, we attempted to compare the gene expression profiles in peripheral blood mononuclear cells among individuals with active TB, LTBI, and healthy conditions. We identified a panel of mRNAs that differed among these groups and subsequent validations with independent samples established the potential use of these gene expression biomarkers for the discrimination of LTBI from active TB in the Taiwanese population.

## Results

### Differentially expressed genes among TB, LTBI, and healthy controls

**Fig. 1:**
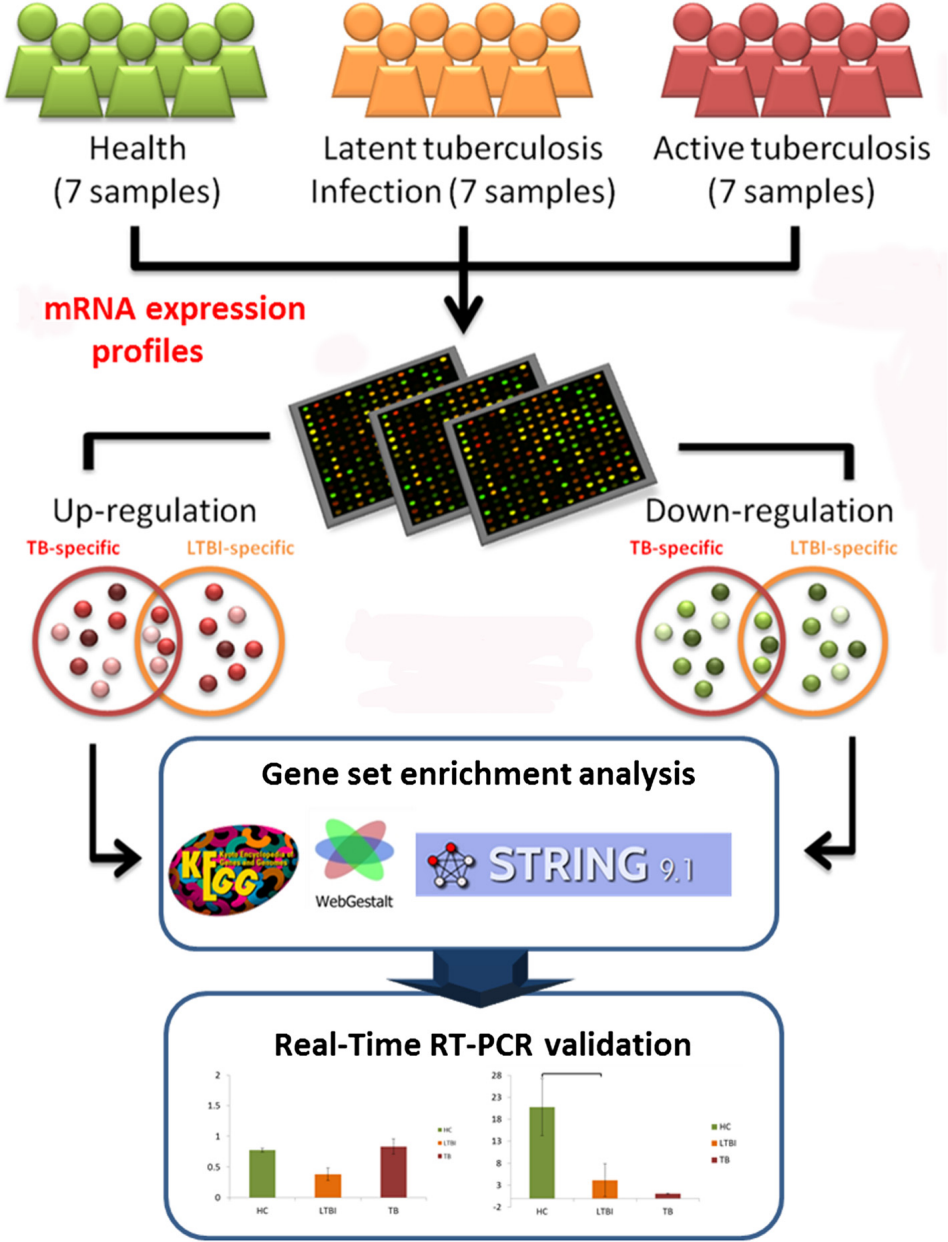
System flow of our analysis

**Fig. 2:**
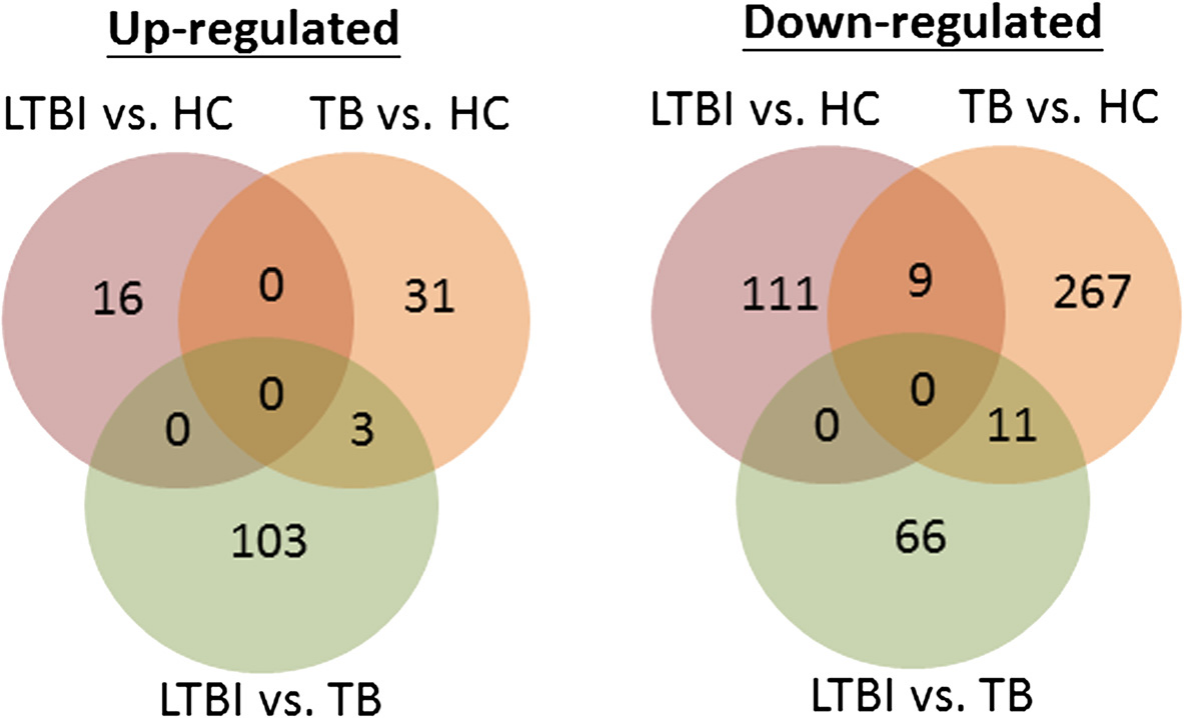
Number of differentially expressed genes among TB, LTBI, and healthy control (HC) group. Significant differential expression is represented by an absolute log_2_ fold change ≥1, FDR < 0.05

To identify candidate genes whose expression levels may differentiate among TB, LTBI, and healthy controls, we followed the workflow as illustrated in Fig. 1. The TB, LTBI, and healthy controls recruited for gene expression profiling did not differ significantly in age (One-way ANOVA: F2,18 = 0.21, *p* = 0.81; Additional file 1). Compared to healthy individuals, 31 and 16 genes were up-regulated in TB and LTBI, respectively (Fig. 2). While a total of 267 genes showed significantly reduced expression in TB patients relative to healthy controls, 111 genes appeared to be expressed at a lower level in those affected with LTBI compared with their healthy counterparts. Between TB and LTBI, 169 genes were differentially expressed, with 103 genes presenting increased abundance and 66 genes exhibiting decreased expression in LTBI relative to TB. Among these differentially expressed genes, three and 11 were also up-regulated and down-regulated, respectively, between LTBI and healthy controls. A list of the differentially expressed genes is provided in Additional file 2.

### Functions, pathways, and interactions associated with the differentially expressed genes

According to the gene set enrichment analysis, genes differentially expressed among TB, LTBI, and healthy controls were over-represented in different GO categories (Table 1). The detailed lists of GO comparisons can be found in Additional files 3, 4, and 5 for LTBI reltiave to healthy controls, TB relative to healthy controls, and LTBI relative to TB, respectively. Compared with healthy controls, TB-specific gene expression profile appeared to be mostly related to leukocyte differentiation, lymphocyte activation, chemokine receptor activity, and regulation of immune response. In contrast, those latently infected with TB and healthy controls showed differing expression in genes belonging to regulation of metabolism, apoptosis, translation, and signal transduction pathways involving MAP kinase phosphatase and protein tyrosine/threonine phosphatase activities. Between the TB and LTBI group, the differentially expressed genes were not only enriched in immune system associated categories such as immune response activation and regulation, as well as natural killer cell and T-cell differentiation, but these genes were also involved in cellular processes like translation, transcription, and mRNA catabolism.

**Table 1:** Gene Ontology categories enriched by differentially expressed genes among TB, LTBI, and healthy control (HC)

TB vs. HC	LTBI vs. HC	LTBI vs. TB
1. Immune response	Regulation of metabolic process	Response to cold
2. Leukocyte differentiation	Regulation of cellular Metabolic process	Immune Response-regulating signaling pathway
3. Immune system process	Regulation of biosynthetic process	Cellular process
4. B cell activation	Apoptotic process	Immune response- activating signal transduction
5. Lymphocyte differentiation	Death	Heterotypic cell-cell adhesion
6. Regulation of immune response	Regulation of gene expression	NK T cell differentiation
7. Positive regulation of response to stimulus	MAP kinase phosphatase activity	Regulation of mRNA catabolic process
8. Lymphocyte activation	Translation factor activity,	Translation regulator activity
9. Leukocyte activation	Translation initiation factor activity	NF-kappaB binding
10. Chemokine receptor activity	Protein tyrosine/threonine phosphatase activity	Translation repressor activity

WebGestalt setting: multiple test adjustment = Benjamini-Hochberg, significance level = top 10 (Benjamini-Hochberg adjusted *p* < 0.05); minimum number of genes for a category = 2

Pathway analysis revealed that relative to LTBI and healthy controls, most genes affected by active TB appeared to be involved in the regulation of immune responses (Table 2). For example, many differentially expressed genes between the TB and healthy control group were mapped to pathways associated with cytokine-cytokine receptor interaction, inflammatory responses such as rheumatoid arthritis, graft-versus-host disease and cancer. In addition, the transcriptional profiles that differed between LTBI and TB showed genes concentrated in apoptosis and signaling pathways involving chemokines, Toll-like receptors, and lymphocytes such as B- and T-cells. On the other hand, for genes differentially expressed between LTBI and healthy control, the most enriched pathway belonged to MAPK signaling cascade, followed by adipocytokine signaling modulated inflammatory response and Toll-like receptor signaling mediated innate immunity. The full lists of pathway comparisons for LTBI reltiave to healthy controls, TB relative to healthy controls, and LTBI relative to TB can be found in Additional files 6, 7, and 8, respectively.

**Table 2:** Pathways enriched by differentially expressed genes among TB, LTBI, and healthy control (HC)

KEGG pathway	ID	Genes
TB vs. HC		
Cytokine-cytokine receptor interaction	04060	*CCR6*, *IL7R*, *CCR7*, *FLT1*, *CCR2*, *TNFSF13B*, *FASLG*, *IL2RB*, *CD27*, *IL23A*, *TGFBR2*
Rheumatoid arthritis	05323	*TLR4*, *IL23A*, *HLA*-*DOB*, *ATP6V0E2*, *FLT1*, *TNFSF13B*
Pathways in cancer	05200	*MYC*, *ITGA6*, *FASLG*, *RASSF5*, *HSP90AA1*, *CASP8*, *TRAF4*, *TRAF5*, *TGFBR2*, *ETS1*
Graft-versus-host disease	05332	*FASLG*, *KLRC1*, *PRF1*, *HLA*-*DOB*
MAPK signaling pathway	04010	*FASLG*, *RRAS2*, *TGFBR2*, *MYC*, *RASA2*, *PRKACB*, *MAP3K1*
LTBI vs. HC		
MAPK signaling pathway	04010	*TNF*, *JUN*, *DUSP2*, *ATF4*, *NR4A1*, *FOS*, *DUSP5*, *DDIT3*, *PDGFRB*, *DUSP1*
Adipocytokine signaling pathway	04920	*POMC*, *TNF*, *STK11*, *CAMKK2*, *NFKBIA*, *SOCS3*
Leishmaniasis	05140	*TNF*, *JUN*, *FOS*, *NFKBIA*
Toll-like receptor signaling pathway	04620	*TNF*, *JUN*, *FOS*, *NFKBIA*
Cytokine-cytokine receptor interaction	04060	*TNF*, *CXCL2*, *TNFSF14*, *PDGFRB*, *OSM*
LTBI vs. TB		
Chemokine signaling pathway	04062	*GSK3B*, *CCR6*, *CXCL16*, *ARRB2*, *PIK3CG*, *NFKBIA*, *PRKACB*
Apoptosis	04210	*CASP8*, *PIK3CG*, *NFKBIA*, *TNFRSF10B*, *PRKACB*
T cell receptor signaling pathway	04660	*GSK3B*, *PTPRC*, *FOS*, *PIK3CG*, *NFKBIA*
Toll-like receptor signaling pathway	04620	*LY96*, *CASP8*, *FOS*, *PIK3CG*, *NFKBIA*
B cell receptor signaling pathway	04662	*GSK3B*, *FOS*, *PIK3CG*, *NFKBIA*

Benjamini-Hochberg adjusted *p* < 0.05

**Fig. 3:**
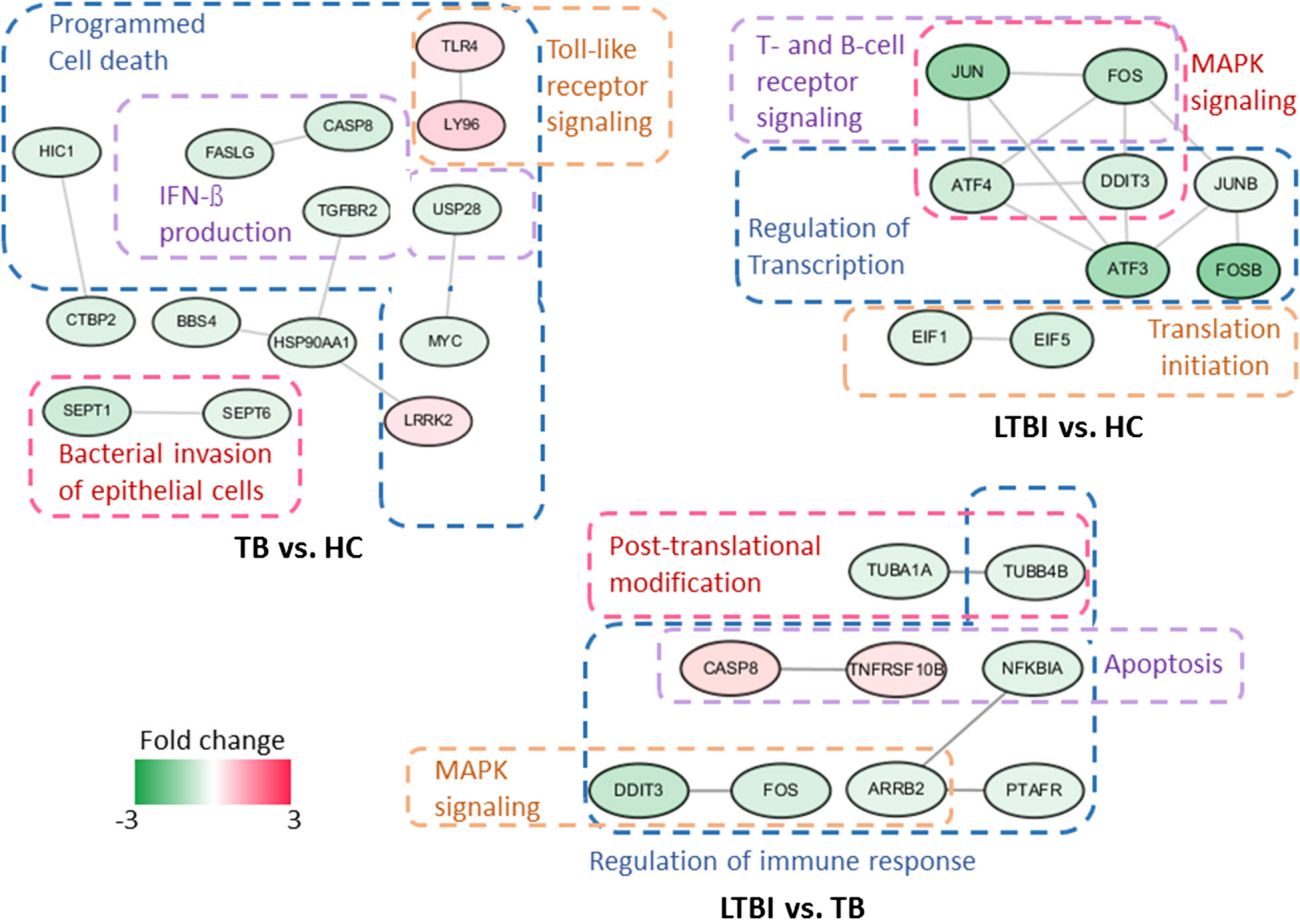
Protein interaction networks of differentially expressed genes among TB, LTBI, and healthy control (HC). Genes are grouped according to their associated pathways and functions

Protein interaction analysis identified specific interaction network modules for active TB, LTBI, and healthy controls. The network modules were grouped according to their GO annotations and have been cross-validated with the STRING database [19, 20] (Fig. 3). Among the genes differentially expressed between LTBI and healthy controls, protein interactions involved in transcriptional regulation (*ATF3*, *ATF4*, *JUNB*, *FOSB*, and *DDIT3*), as well as translation initiation (*EIF1* and *EIF5*) appeared to be the most important. Whereas proteins that regulate interferon-beta production (*LY96* and *TLR4*), apoptotic signaling (*HSP90AA1*, *LRRK2*, *TGFBR2*, FASLG, CASP8,), bacterial invasion (*SEPT1* and *SEPT6*), and Wnt signaling pathway (*HIC1* and *CTBP2*) seemed to represent the underlying variations between TB and healthy controls, the differences between TB and LTBI might be contributed by proteins that modulate transcription (*FOS* and *DDIT3*), phagosome formation (*TUBA1A* and *TUBB4B*), autophagy (*CASP8* and *TNFRSF10B*), and interferon-gamma signaling (*ARRB2*, PTAF*R*, *NFKBIA*). Additional files 9, 10, and 11 contain detailed lists of enriched protein interaction modules associated with genes differentially expressed in LTBI relative to healthy controls, TB relative to healthy controls, LTBI relative to TB, respectively.

### Validation of differentially expressed candidate biomarkers

Many of the identified differentially expressed genes among the TB, LTBI, and healthy control group have also been implicated in TB pathology by other groups. However, as indicated by our analysis, several of these genes play roles in other infections, inflammatory diseases, cancers or even common cold. For real-time RT-PCR validation, we selected genes that are known to be expressed in the lungs and showed clear differences in transcript abundance (fold change ≥1) in at least one of the comparisons; that is, TB versus healthy controls, LTBI versus healthy controls, or LTBI versus TB. Additional volunteers were recruited for gene expression validation. To avoid overlaps with other respiratory tract infections, we chose three differentially expressed genes that may not be directly involved in mediating the immune and inflammatory responses against common respiratory infections. These genes were *NEMF* (nuclear export mediator factor), *ASUN* (asunder spermatogenesis regulator), and *DHX29* (DEAH (Asp-Glu-Ala-His) box polypeptide 29). Then, we selected *PTPRC* (protein tyrosine phosphatase, receptor type, C) or CD45, an estalished marker of active TB [21], as a reference standard.

**Fig. 4:**
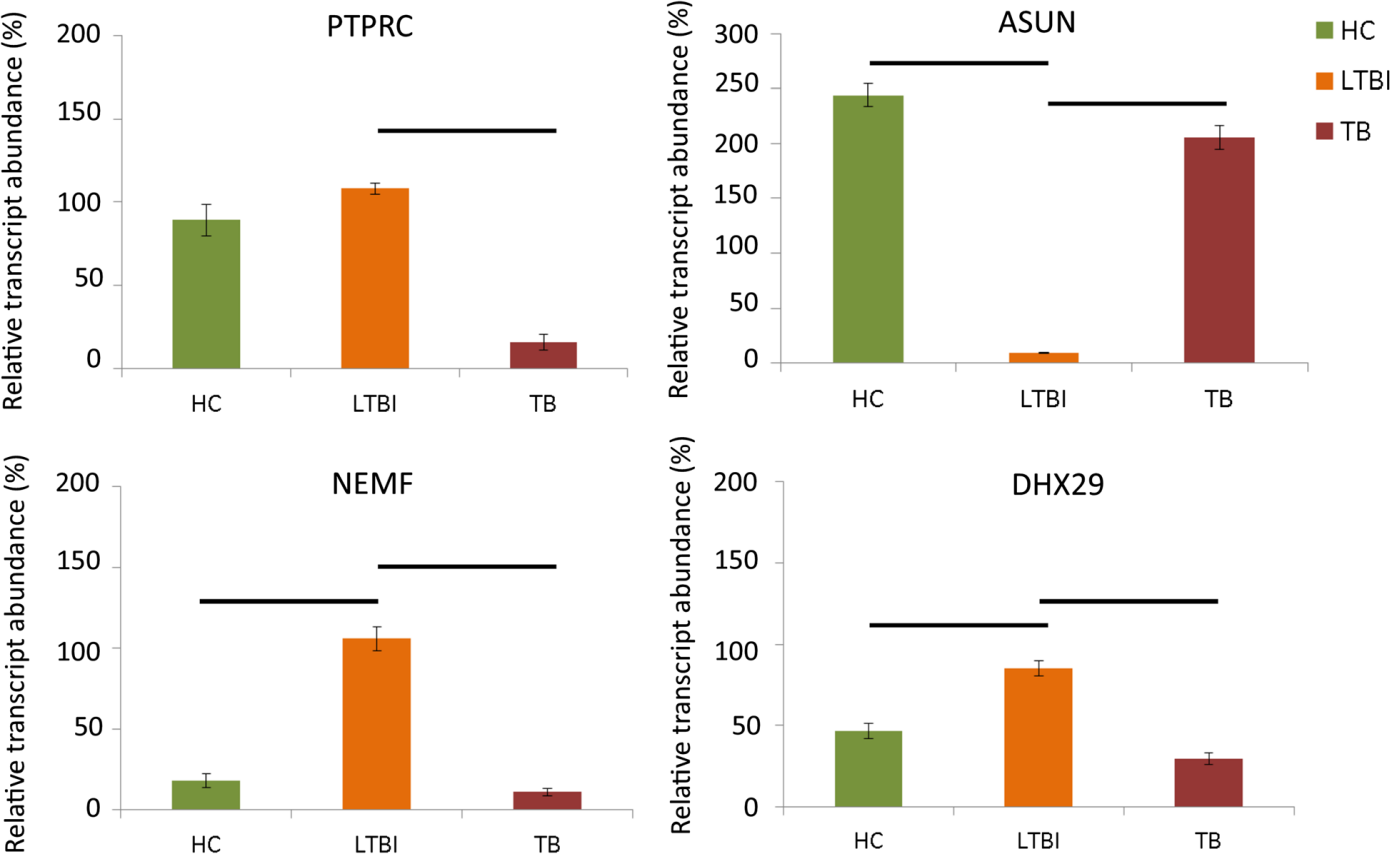
Validation of four differentially expressed genes among TB, LTBI, and healthy controls (HC). Statistical significance (*p* < 0.05) is represented by a *horizontal bar*

**Fig. 5:**
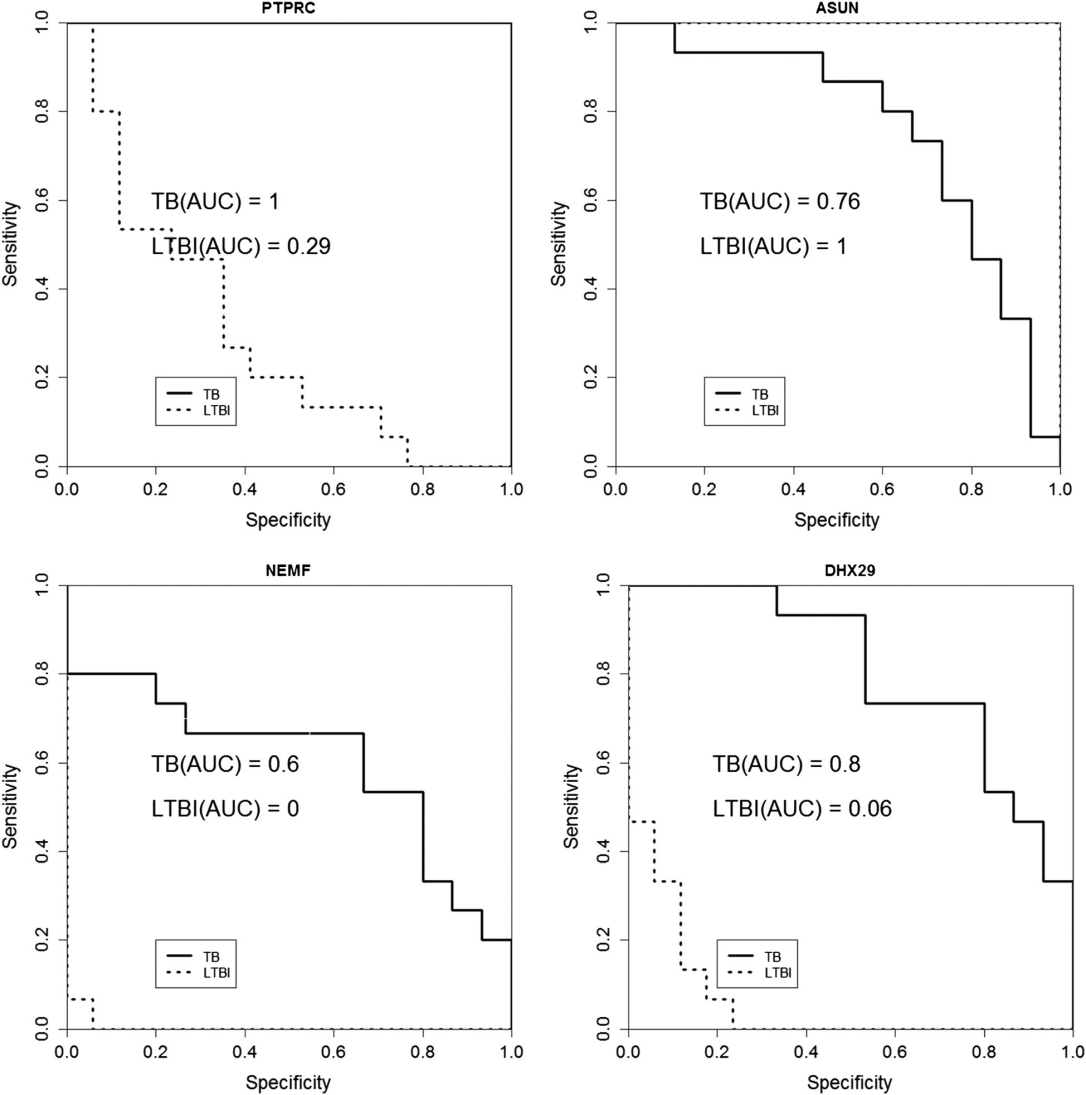
ROC analysis of four differentially expressed genes among TB, LTBI, and healthy controls (HC). AUC represents the area under the curve

Though the independently recruited participants differed significantly in age (Additional file 1), RT-PCR results successfully verified the array observations in the gene expression array experiment (Fig. 4), indicating that age might not have been a major factor. Subsequent ROC analyses confirmed that *PTPRC* expression may be able to detect active TB, while *ASUN* could discriminate TB or LTBI from healthy individuals (Fig. 5). Other than *PTPRC*, the transcript abundance of *DHX29* could also distinguish the differences between TB and healthy controls. In contrast, *NEMF* did not demonstrate to be a good discriminatory biomarker.

Finally, to assess the ability of *PTPRC*, *DHX29*, and *ASUN* in classifying TB, LTBI, and healthy individuals, as a proof of concept experiment, we tested the performance of classification models built with the candidate biomarkers using a sample size of 17 LTBI, 15 TB, and 15 healthy individuals. We utilized four classifiers: decision tree, random forest, support vector machine (SVM), and Naïve Bayes. As evaluated by a 5-fold cross-validation approach, the accuracy, sensitivity, and specificity of the models constructed with single candidate genes were relatively low compared to those built using a combination of biomarkers (Additional file 12 for single gene models; Table 3 for hybrid models). The Naïve Bayes-based model, which was constructed with the expression levels of *PTPRC*, *DHX29*, and *ASUN* as the selected features, yielded the best performance (Table 3).

**Table 3:** Performance of diagnostic support models constructed using combinations of candidate biomarkers with various classifiers

Features	Classifier	Accuracy	Sensitivity	Precision	AUC
*PTPRC*+*ASUN*	Decision tree	91.49 %	91.5 %	97.7 %	0.943
*PTPRC*+*ASUN*+*DHX29*	Random Forest	93.62 %	93.6 %	93.6 %	0.982
*PTPRC*+*ASUN*+*DHX29*	SVM	95.74 %	95.7 %	96.2 %	0.969
*PTPRC*+*ASUN*+*DHX29*	Naïve Bayes	97.87 %	97.9 %	98 %	0.979

Sensitivity: TP/(TP+FN); Precision: TP/(TP+FP); performance was evaluated by 5-fold cross-validation

## Discussion

TB is a serious health threat among the young, elderly, and immunocompromised. Variations in the transcriptional profile of human peripheral blood mononuclear cells in the presence of *Mtb* infection are complex and can be attributed to multiple factors, including age [22], genetic background [14, 23], and study designs. Therefore, identifying distinct genetic signatures of active TB, LTBI, and healthy individuals that is population-specific becomes a challenging task.

In the current study, we examined the transcriptomes of active TB, LTBI, and healthy individuals, and uncovered specific molecular markers and pathways associated with each group. We found that there were more genes showing differential expression between the TB group and healthy controls as compared with LTBI versus healthy controls. GO and pathway enrichment analyses revealed that the transcriptional profiles of TB individuals differed from those of healthy controls in immune system processes such as leukocyte and lymphocyte activation, differentiation, chemokine receptor activity. In contrast, although immune pathway alterations were indeed observed in individuals with LTBI at the transcript level, metabolic processes in these individuals also differed from the healthy controls. On the other hand, between TB and LTBI patients, the most important genes seemed to be mediators of inflammation, immune system responses, and apoptosis.

Our results support findings from other studies in that infection with *Mtb* triggers a relay of inflammatory signals and immune responses from the host. Upon entry, *Mtb* are recognized by various host receptors, including Toll-like receptors (TLRs) and nucleotide-binding oligomerization domain-like receptors that are expressed on immune cells [24]. This host-pathogen interaction initiates a cascade of inflammatory responses, whereby alveolar macrophages produce cytokines and chemokines to alert the host of the infection [25]. In response to this signal, T- and B-lymphocytes aggregate around the infected macrophages to form granulomas, a microenvironment to prevent bacterial spread and an isolated region for the lymphocytes to invoke apoptosis of the infected macrophages [26]. The bacteria have also evolved tactics to avoid this fate. After being engulfed by macrophages, the bacteria multiply in the phagosome, causing macrophage necrosis and allowing for their escape from the host defenses to infect other cells [25]. This is evident in our results in which a lot of the differences between TB and healthy controls were associated with mediators of immune responses.

In particular, our protein interaction analysis suggests that Toll-like receptor 4 (TLR4) may mediate the host defense against *Mtb*. It is known that TLR4 is important in modulating the balance between apoptosis and necrosis in *Mtb*-infected macrophages [27]. Our analysis showed that TLR4 may interact with LY96 (lymphocyte antigen 96) and S1PR1 (Sphingosine-1-phosphate receptor 1) in TB patients. LY96 is responsible for establishing the binding between the lipopolysaccharide on the bacterial cell membrane and TLR4 on the host cell surface, thus activating a series of immune responses in infected individuals [28].

The bacterial strain *Mtb* has quite a few tricks to blunt the bactericidal mechanisms of infected macrophages so as to promote their persistence in the host. In latent TB infection, the bacteria manipulate the host antigen presentation processes and enter a non-replicating state [29]. As a result, the bacteria remain dormant inside the phagosomes, and the granuloma became their safe hideout. This is evident in our observation that TB and LTBI differed not only in the regulation of immune responses, but also in the modulation of phagosome, autophagy and apoptosis. In fact, several apoptosis-associated molecules, such as decoy receptor 3, prostaglandin 2, and lipoxin, have been shown to correlate significantly with the status of *Mtb* infection [30, 31].

Compared with healthy controls, genes involved in cell cycle control seemed to be differentially regulated in individuals with LTBI. Our interaction analysis showed that this difference may be attributable to the interactions between mediators of the MAPK (mitogen-activated protein kinase) signaling pathway, such as JUN (Jun proto-oncogene), JUNB (Jun B proto-oncogene) and FOSB (FBJ murine osteosarcoma viral oncogene homolog B), as well as regulators of translation. MAPK signaling is one of the pathways responsible for modulating the host’s innate immunity [32]. In fact, MAPK activation is important for inducing the expression of genes involved in inflammatory responses, but inactivation of MAPK activity is also important to prevent host tissue damage [32]. On the other hand, our interaction analysis also indicated that translational regulation represent important differences between LTBI and healthy individuals. It is known that bacteria can influence host tranlation in order to immobilize host defenses and promote their own survival [33]. Therefore, our finding supports that MAPK signaling and tranlsational control may underlie the differences between LTBI and healthy conditions.

In addition, alterations in the expression of genes involved in metabolic processes seemed to be a major difference between the LTBI group and healthy controls. Metabolism-related manifestations are known to be associated with TB [34]. It has been demonstrated that *Mtb*-infected macrophages would become lipid-loaded (foamy) in the granuloma, and the fatty acids accumulated as triacylglycerol represent the vital source of energy for dormant *Mtb* [35]. Also, certain immune-endocrine-metabolic alterations are thought to exist in TB patients, though molecular studies have yet to reach a consensus as to which molecules are the major players in this process [36–39].

To date, most identified biomarkers for active TB and LTBI are related to inflammatory and immune responses triggered by the pathogen infection [14, 18]. However, TB may be comorbid with various communicable diseases including influenza, bacterial pneumonia, HIV, syphilis, leishmaniasis, as well as non-communicable disorders such as diabetes, alcohol-related diseases, chronic obstructive pulmonary disease, coronary artery disease, cancer, etc. [40]. In fact, our gene set enrichment analysis mapped four genes, *TNF*, *JUN*, *FOS*, *NFKBIA*, that were differential expressed between LTBI and healthy controls to leshimaniasis-related pathways.

To avoid finding biomarkers that overlap with other diseases, we chose to verify differentially expressed genes that have not been identified as TB or LTBI biomarkers in independently recruited samples. We also included an established active TB marker, *PTPRC*, as a test reference. As a result, *ASUN*, *DHX29*, and *NEMF* were successfully confirmed to be differentially expressed among active TB, LTBI, and healthy individuals. Subsequent ROC analysis revealed the potential of *PTPRC*, *ASUN*, and *DHX29* in discriminating among TB, LTBI, and healthy conditions. Further classification experiments also indicated that combinining the three canidate biomarkers may be more effective in achieving accurate identification of the different disease states.

To be a biomarker, the gene should to be related to the pathogenesis of the disease. However, since functional analysis was not performed in this study, we try to postulate how these candidate genes may be involved in TB pathology based on their known functions. Among the validated genes, PTPRC is an essential regulator of host immune response through the modulation of T- and B-cell receptor signal transduction [41]. In the guinea pigs, *PTPRC* expression appeared to increase after exposure to *Mtb* and decrease after the infection persisted for a longer period of time [42]. This is in line with our observation that *PTPRC* transcript level was decreased in active TB compared to LTBI and healthy state. *ASUN* is critical for the regulation of mitotic cell cycle [43]. As the number of T-cells can determine whether an individual is susceptible to *Mtb* infection or active TB [44], we suspect that the high level of *ASUN* expression in LTBI relative to the active TB and healthy controls may be associated with T-lymphocyte differentiation or proliferation. *NEFM* is a nuclear export mediator that have been implicated as a tumor suppressor in lung cancer [45]. Nuclear export is an important process for the regulation of autophagy [46]. A recent in vivo study performed in mice suggested that autophagy can suppress the progression towards active TB by inhibiting *Mtb* growth [47]. Finally DHX29 is a helicase protein that participates in translation initiation, and its down-regulation has an inhibitory effect on cancer growth [48], which may be related to the altered cellular processes in TB.

Note that our study is limited by the small sample size and lack of functional studies to determine the roles that the identified candidate biomarkers play in the pathogenesis of TB. This might have also been the reason that we did not observe more genes associated with the TLR signaling pathway, an established mechanism underlying *Mtb* recognition [49]. As a result, this pathway was not the most enriched in any of the comparisons. Equally likely is that the changes in *TLR* genes might be dependent on treatment duration. It has been demonstrated that the increase in *TLR4* expression level appeared to be more significant compared to *TLR2* after 1 month of treatment in TB patients when compared with healthy controls [50]. In our study, samples were collected at the time of diagnosis. More evident changes might be observed if we had examined the temporal expression profiles of TB and LTBI patients during treatment. Also, our experiments were focused on blood cells, instead of lung tissues. Therefore, the results are more relevant to biomarkers associated with TB and LTBI, and perhaps indirectly related to the pathology of the disease in the lungs. Moreover, BCG is known to have an effect on gene expression [51, 52]. In Taiwan, BCG vaccine should be administered to every newborn; therefore, all of our study participants have been inoculated with BCG. This may make our finding specific to not only the Taiwanese people, but also to those who have received this vaccine. Finally, despite the fact that evaluation of the biomarkers yielded relatively good results, due to the limited sample size and the variable prevalence of TB and LTBI in different seasons, we could not eliminate the possibility that other diseases or environmental factors may affect the effectiveness of the candidate biomarkers.

## Conclusions

In conclusion, we have performed a genome-wide gene expression study comparing the transcriptional profiles among TB and LTBI patients, as well as healthy controls. Gene set enrichment analyses have identified specific biological processes and pathways associated with genes that are differentially expressed among these groups. We have uncovered novel discriminatory biomarkers for TB and LTBI. Moreover, we have demonstrated, as a proof of concept, that the expression levels of *PTPRC*, *ASUN*, and *DHX29* may be used as diagnostic biomarkers. Validation of the diagnostic support system in a larger sample size would help confirm the discriminative potential of these biomarkers and facilitate the development of a cost-effective and sensitive molecular diagnostic platform for TB.

## Methods

### Clinical sample collection

The analytical flow of our study is illustrated in Fig. 1. All procedures were reviewed and approved by the Institutional Review Board of Taoyuan General Hospital, Ministry of Health and Welfare, Taoyuan, Taiwan. Written informed consents were obtained from all participants. For those who had reduced ability to consent (including minors), the carers or guardians gave written informed consents on behalf of these participants. Eligibility for entry into the study was based on clinical signs and symptoms of Mtb infection. All participants were interviewed and examined by a physician. Each subject received the sputum smear test, T-SPOT TB test, and took a chest radiograph. TB subjects were those who showed clinical signs of TB, in addition to having been tested positive on all tests. LTBI subjects were recruited from close contacts (>8 h/day for a total of >40 h of close contact) with active TB patients, tested negative on the smear test, positive on the T-SPOT TB test, appeared normal on their chest radiographs, and exhibited no clinical evidence of active TB. Healthy controls were individuals who had not been in close contact with TB or LTBI patients, obtained negative results on all tests and showed no clinical signs of TB or LTBI. Individuals with allergic diseases, diabetes, cancer, immune-compromised conditions, and co-infections with any types of infectious diseases were excluded. In total, seven healthy individuals, seven patients with active TB, as well as seven subjects with LTBI, were included in the microarray experiment. Additional participants (15 TB, 17 LTBI, and 15 healthy individuals) were recruited as independent testing samples for validation of the expression profiling results. Age and gender information are provided in Additional file 1. In Taiwan, every newborn must be inoculated with BCG; therefore, the BCG inoculation status for every participant was positive.

### RNA isolation

RNA was isolated from peripheral blood mononuclear cells (PMBC). The quality of RNA was determined by an optical density (OD) 260/280 ratio ≥1.8, and OD 260/230 ratio ≥1.5 on a spectrophotometer, in addition to the intensity of the 18S and 28S rRNA bands on a 1 % formaldehyde-agarose gel. RNA quantity was detected by a spectrophotometer. RNA integrity was examined on an Agilent Bioanalyzer. RNA with an RNA integrity number (RIN) ≥7.0 and 28S/18S >0.7 was subjected to microarray analysis.

### Gene expression analysis

RNA samples were subjected to Human OneArray® v6 (Phalanx Biotech, Hsinchu, Taiwan). Data were analyzed with the Rosetta Resolver System software (Rosetta Biosoftware, USA). The criteria for identifying differentially expressed genes were: 1) an absolute log2 fold change ≥1; 2) a false discovery rate of <0.05; 3) an intensity difference of >1000 between two samples under comparison; 4) an individual intensity of >500. Genes showing significant differential expression were categorized into TB versus healthy controls, LTBI versus healthy controls, and LTBI versus TB. Our gene expression records can be found on the Gene Expression Omnibus under the accession number GSE62525.

### Bioinformatics analysis

Differentially expressed genes were used as input for a series of bioinformatics analyses performed with the WEB-based GEne SeT AnaLysis Toolkit (WebGestalt) [53, 54]. WebGestalt is an open analytical platform that integrates gene ontology (GO) [55], KEGG [56], WikiPathway [57], protein interaction networks, microRNA binding sites and transcription factor targets [58], as well as cytogenetic band information, for a variety of enrichment analyses. The GO, KEGG, and protein interaction module tools were utilized to analyze the differentially expressed genes. Multiple testing bias was adjusted by a Benjamini-Hochberg threshold of *p* < 0.05. The enriched protein interaction network modules in each transcriptional profile were grouped according to their GO annotations or associated pathways. Experimentally confirmed interactions were cross-validated with the STRING database (v9.1) [19, 20] with a confidence level of 0.7 as the paratmeter setting. Visualization of the interaction networks was achieved using Cytoscape version 3.2.1 [59].

### Real-time RT-PCR validation

The four differentially expressed genes selected for real-time RT-PCR validation were *PTPRC*, *ASUN*, *NEMF*, and *DHX29*. Total RNA from PBMC was extracted using TRIzol (Invitrogen, Carlsbad, CA, USA) from 17 TB, 15 LTBI, and 15 healthy individuals. Each extracted RNA sample was reversely transcribed using the First Strand cDNA Synthesis kit (Roche, Boulder, CO, USA) according to the manufacturer’s instructions. Each cDNA sample was amplified with the FastStart Universal SYBR Green reagent (Roche, Mannheim, Germany) on the StepOnePlusTM Real-Time PCR system (Applied Biosystem, CA, USA). Briefly, the reaction conditions consisted of 10 ng of cDNA and 0.25 μM of primers in a final reaction volume of 20 μl in SYBR Green mixture. Each reaction was initiated with 10 min at 95 °C, followed by 40 cycles consisting of denaturation at 95 °C for 15 s, annealing at 60 °C for 1 min, and extension at 72 °C for 30 s. For each reaction, the β-actin gene was used as an endogenous control to normalize each sample. Primer sequences for each gene are listed in Additional file 13. The relative expression of each gene was compared using the 2-ΔΔ CT method and all experiments were run in triplicates and repeated three times. Differences in expression among TB, LTBI, and healthy controls were evaluated with one-way ANOVA followed by Tukey’s post hoc test in SPSS 18.0 (IBM Corporation, NY, USA). A *p*-value of <0.05 was regarded as statistically significant. A receiver operating characteristic (ROC) curve analysis was performed in the R statistical environment (3.1.1) to assess the specificity and sensitivity of each validated biomarker.

### Construction of a diagnostic support model

We wished to test if the candidate biomarkers could be integrated in a diagnostic support system. As a proof of concept, we used the expression levels of *PTPRC*, *ASUN*, and *DHX29* as features to build classification models based on the 57 volunteers who donated their blood samples for RT-PCR validation. Due to its low discriminating abiliy as evaluated by ROC analysis, *NEMF* was excluded. Experiments were conducted in LibSVM (version 3.12) [60] and WEKA, or Waikato Environment for Knowledge Analysis (version 3.6.5) [61]. Features were selected based on the previous ROC analysis results. The selected classifiers included the C4.5 decision tree algorithm [62], support vector machine (SVM) [63], Naïve Bayes [64, 65], and the random forest algorithm [66]. Models were built with single genes or a combination of the selected genes. Performance of each model was evaluated by 5-fold cross-validation.

## Additional files

Additional file 1:
**Average age and gender distribution of the participants** (XLSX 11 kb) Additional file 2:
**List of differentially expressed genes among the TB, LTBI, and healthy individuals.** (XLSX 19238 kb) Additional file 3:
**GO enrichment of genes differentially expressed in LTBI relative to healthy controls.** (XLSX 111 kb) Additional file 4:
**GO enrichment of genes differentially expressed in TB relative to healthy controls.** (XLSX 69 kb) Additional file 5:
**GO enrichment of genes differentially expressed in LTBI relative to TB.** (XLSX 66 kb) Additional file 6:
**Pathway enrichment of genes differentially expressed in LTBI relative to healthy controls.** (XLSX 19 kb) Additional file 7:
**Pathway enrichment of genes differentially expressed in TB relative to healthy controls.** (XLSX 20 kb) Additional file 8:
**Pathway enrichment of genes differentially expressed in LTBI relative to TB.** (XLSX 20 kb) Additional file 9:
**Enriched protein interaction modules associated with genes differentially expressed in LTBI relative to healthy controls.** (XLSX 14 kb) Additional file 10:
**Enriched protein interaction modules associated with genes differentially expressed in TB relative to healthy controls.** (XLSX 15 kb) Additional file 11:
**Enriched protein interaction modules associated with genes differentially expressed in LTBI relative to TB.** (XLSX 20 kb) Additional file 12:
**Performance of diagnostic support models constructed using individual candidate biomarkers with various classifiers.** (XLSX 11 kb) Additional file 13:
**RT-PCR Primer for the selected differentially expressed genes.** (XLSX 11 kb)
